# Complementary and Alternative Medicine in Cancer Prevention and Therapy

**DOI:** 10.1155/2015/639372

**Published:** 2015-04-01

**Authors:** Peng Cao, Senthamil R. Selvan, Esra Küpeli Akkol, Ning Wang, Hongjun Yang, Xiaolan Cheng

**Affiliations:** ^1^Laboratory of Cellular and Molecular Biology, Jiangsu Province Institute of Traditional Chinese Medicine, Nanjing 210028, China; ^2^Department of Medical Oncology, Thomas Jefferson University, Philadelphia, PA 19107, USA; ^3^Department of Pharmacognosy, Faculty of Pharmacy, Gazi University, Etiler, 06330 Ankara, Turkey; ^4^Department of Oncology, Luxembourg Institute of Health, 84 Val Fleuri, 1526 Luxembourg, Luxembourg; ^5^Institute of Chinese Materia Medica, China Academy of Chinese Medical Sciences, China

Although there have been great achievements in the battle against cancer over the past decades, cancer is still the leading cause of death in developing countries like China. Complementary and alternative medicine (CAM), especially traditional Chinese medicine (TCM), usually having good clinical tolerability, is applied as an adjuvant therapy to treat cancer in China based on TCM or modern pharmacological theories. In the west, CAM has increasingly become popular in cancer patients. It is estimated that the United States National Cancer Institute (NCI) spends around $120 million each year on complementary and alternative medicine related research projects. However, a fact is that although a lot of CAMs are widely used clinically, most evidence supporting their use came from poorly reported published clinical studies or studies with questionable methodology. Call for high quality evidence is a mission of this special issue. This issue on CAM for treatment or prevention of cancer compiles 13 exciting manuscripts, most of which reporting the efficacy and possible mechanisms of action of CAM or active constituents on cancer prevention and/or therapy in clinical and experimental levels.

S. S. M. Fong et al. contributed high quality evidence about Tai Chi Qigong Training for balance performance in irradiated survivors of nasopharyngeal cancer by a well-designed, well-conducted, and well-reported randomized controlled trial. Totally 120 senior adults participated in the study voluntarily. In this cross-sectional exploratory study, they have done very well on the ethics issues including protocol approval by ethical committee, properly informed consent, and high quality methodological issues, including the randomization method and allocation procedure, the recruitment of patients, the data management, the analysis method of results. All of the procedures were conducted according to the Declaration of Helsinki. This transparency makes readers confident to trust the result of the study. This is the first study to show that participating survivors of NPC had inferior OLS balance performances in all of the visual and supporting surface conditions compared with age-matched healthy counterparts. TC Qigong might be a potential rehabilitation exercise to improve the somatosensory function and single-leg standing balance performance of survivors of NPC. The shortcoming of this study is that it uses a convenience sample which may have introduced a self-selection bias that may threaten the internal validity of the study. In addition, homogenous subject group, small sample size, and OLS clinical test for balance performance assessment may limit the generalizability of results. However, we still believe that this study and its report could be a good evidence for efficacy of CAMs.

This edition also includes a review that discusses the possibility of TCM as maintenance therapy for advanced nonsmall cell lung cancer. The review concludes that TCM as maintenance therapy can improve the QOL and prolong the PFS of advanced NSCLC patients. Besides, TCM can be applied for NSCLC patients not limited in population selection. However, there are only small sample clinical trials about TCM as maintenance therapy for advanced NSCLC. More large-scale trials of TCM as maintenance therapy for advanced NSCLC are expected.

For experimental study, A. Movahedi et al. investigated the capability of the decoction of* Teucrium polium* L. from Lamiaceae family to protect liver cells against hepatocellular carcinoma in carcinogenesis-induced animal model. After 28 weeks of treatment, they found that serum biochemical markers (ALT, AST, AFP, GGT, ALP, HCY, TNF-*α*, *α*2MG, and CBG), total antioxidant status, liver lesion, and glucocorticoid activity were all regulated auspiciously by* Teucrium polium* L. Y. Lin et al. studied that elemene, a compound found in an herb used in traditional Chinese medicine, has the effect of protecting cancer cells from death either in apatinib induced nutrient deficient environment or in serum-free induced starvation. Further data on the mechanism study revealed that elemene induced protective autophagy and prevented human hematoma cancer cells from undergoing apoptosis.

In addition to the mentioned papers above, other studies included in this issue provide sufficient scientific evidence from CAM research to clarify their mechanism of action and demonstrate their efficacy and safety. Through the rigorous researches, the benefits of CAM therapies will be highlighted, and this will support the clinical use of CAMs and help integrating CAM into the mainstream medicine. We hope that this special issue informs us about the rationale use of CAM in cancer prevention and therapy. We also hope that the papers included in this issue play a role in reflecting the recent advancement in the field of CAM.

*Peng Cao* *Peng Cao* *Senthamil R. Selvan* *Senthamil R. Selvan* *Esra Küpeli Akkol* *Esra Küpeli Akkol* *Ning Wang* *Ning Wang* *Hongjun Yang* *Hongjun Yang* *Xiaolan Cheng* *Xiaolan Cheng*

